# Pilot experience of [^161^Tb]Tb-PSMA-617 RLT in mCRPC patients after conventional PSMA RLT within a prospective registry

**DOI:** 10.7150/thno.115831

**Published:** 2025-08-16

**Authors:** Florian Rosar, Caroline Burgard, Christine Petrescu, Arne Blickle, Mark Bartholomä, Stephan Maus, Moritz B. Bastian, Tilman Speicher, Andrea Schaefer-Schuler, Samer Ezziddin

**Affiliations:** Department of Nuclear Medicine, Saarland University - Medical Center, Homburg, Germany.

**Keywords:** terbium-161, ^161^Tb, Tb-161, PSMA, prostate cancer, mCRPC, RLT, radioligand therapy

## Abstract

*Rationale:* The radionuclide ^161^Tb is an increasingly discussed potential candidate for radioligand therapy (RLT). Through the considerable emitted amount of low-energy Auger and conversion electrons, ^161^Tb offers physical advantages over the commonly used ^177^Lu, resulting in higher locally absorbed doses. In this study, we present initial experience with [^161^Tb]Tb-PSMA-617 RLT across different clinical settings following initial PSMA RLT.

*Methods:* The study involved n=18 patients with metastasized castration-resistant prostate cancer (mCRPC) participating in a prospective registry (NCT04833517) and receiving [^161^Tb]Tb-PSMA-617 after initial PSMA RLT with established radionuclides (^177^Lu, ^225^Ac). In total 47 cycles of [^161^Tb]Tb-PSMA-617 RLT were administered with a median of 3 cycles (1 - 4 cycles) per patient. The mean administered activity of ^161^Tb per cycle was 6.2 ± 0.8 GBq, the mean cumulative activity was 16.1 ± 4.9 GBq. Outcome was evaluated by biochemical and molecular imaging response, progression-free survival (PFS), and overall survival (OS). Adverse events were assessed by '*Common Terminology Criteria for Adverse Events*' (CTCAE v.5.0) grading system.

*Results:* In the heterogeneous cohort of patients previously experiencing insufficient response or progression post RLT with [^177^Lu]Lu-PSMA-617, or even after ^225^Ac augmentation, biochemical and molecular imaging response rates were 38.9% and 44.4%, median PFS and OS 3.5 and 11.3 months, respectively. The best response and outcome were observed in patients who initially responded to [^177^Lu]Lu-PSMA-617 RLT. The majority of all post therapeutically recorded adverse events were mild or moderate (CTCAE °1 or °2); higher grades (CTCAE °3 or °4) were rarely observed (2 cases of thrombocytopenia, 4 cases of anemia and 4 cases of renal impairment). No treatment discontinuation due to therapy related adverse events was recorded.

*Conclusion:* These pilot results confirm ^161^Tb as a promising radionuclide for PSMA RLT and suggest [^161^Tb]Tb-PSMA-617 as a potential effective and safe treatment option even in the advanced mCRPC setting after multi-line systemic therapies including standard PSMA RLT. Larger studies are warranted to confirm and extend this initial experience and clinical trials even in earlier CRPC settings appear promising based on our initial impression of this radionuclide-based novelty in PSMA RLT.

## Introduction

Prostate cancer (PC) is currently the second most commonly diagnosed cancer among men worldwide and is simultaneously associated with a high mortality rate 1,2. A considerable number of patients eventually progress into the stage of metastasized castration-resistant prostate cancer (mCRPC), characterized by its resistance to chemical castration with androgen deprivation therapy (ADT) 3-5. Nonetheless, several treatment options can be applied such as novel androgen axis drugs (NAAD) 6,7, chemotherapy 8,9, bone-targeting ^223^Ra treatment 10, and poly ADP ribose polymerase (PARP) inhibitors 11. Despite the well-known beneficial effects of these therapies, the occurrence of primary or acquired resistance is challenging. A favorable therapy option for those patients showing a resistance to other mCRPC treatments is the targeted radionuclide therapy, in particular the prostate-specific membrane antigen (PSMA) targeting radioligand therapy (RLT) 12, using the beta emitter ^177^Lu. The administration of [^177^Lu]Lu-PSMA-617 to patients with mCRPC was approved by the EMA and FDA 13,14 and was shown to be effective, safe and tolerable regarding side effects by a variety of studies 15-19. Besides ^177^Lu, different nuclides are currently under investigation, especially alpha emitters such as ^225^Ac or Auger emitters such as ^161^Tb. PSMA RLT with ^225^Ac has already been established as monotherapy and also as tandem therapy in combination with ^177^Lu 20-22. This therapy showed promising anti-tumor effect, but also relevant side effects: e.g. a recently published systematic review and meta-analysis reported a pooled rate of any-grade xerostomia in 84% of patients receiving [^225^Ac]Ac-PSMA monotherapy and a therapy discontinuation due to xerostomia in 5% of cases 23. The radionuclide ^161^Tb has similar physical decay characteristics to ^177^Lu. A detailed summary of the physical properties of both nuclides is provided in the supplementary material (**Table S1**). Both emit β^-^ particles with comparable energies (^177^Lu: 133 keV vs ^161^Tb: 154 keV) and decay with a comparable half-life (^177^Lu: 6.647 days vs ^161^Tb: 6.906 days). While both nuclides are β^-^ emitters, a possible advantage of ^161^Tb compared to ^177^Lu arises from the higher proportion of emitted low-energy conversion and Auger electrons. Auger electrons are characterized by an ultra-short tissue range (< 500 nm), resulting in a relatively high linear energy transfer (LET; 4-26 keV/µm). As a result, higher local dose densities are provided, which are presumably associated with a favorable anti-tumor effect 24. ^161^Tb showed promising results in pre-clinical studies as well as in first small sample clinical reports 25,26. This study aims to present pilot experience, analyzing the outcomes and safety in the largest cohort of patients with mCRPC up to date across different clinical settings following conventional PSMA RLT.

## Methods

### Patients and ethics

The present study included a total of n = 18 patients with mCRPC receiving [^161^Tb]Tb-PSMA-617 and participating in the '*prospective registry to assess outcome and toxicity of targeted radionuclide therapy in patients with mCRPC in clinical routine*' (REALITY Study; NCT04833517) from which they were selected consecutively. All patients received [^161^Tb]Tb-PSMA-617 RLT after initial PSMA RLT with established radionuclides (^177^Lu, ^225^Ac). Intense PSMA expression - defined as tumoral uptake greater than that of the liver on [^68^Ga]Ga-PSMA-11 PET/CT - was a prerequisite for RLT.

In three different clinical settings [^161^Tb]Tb-PSMA-617 was applied:

I) n = 10 patients had undergone [^177^Lu]Lu-PSMA-617 RLT with initial response and post-RLT progression before starting [^161^Tb]Tb-PSMA-617 RLT.

II) n = 3 patients received initial [^177^Lu]Lu-PSMA-617 RLT with insufficient response (progression or stable disease), followed by a switch to [^161^Tb]Tb-PSMA-617.

III) n = 5 patients received [^177^Lu]Lu-PSMA-617 and [^225^Ac]Ac-PSMA-617 RLT with insufficient response, preceding [^161^Tb]Tb-PSMA-617 RLT.

All patients were heavily pre-treated, including treatments preceding initial PSMA RLT, detailed patient characteristics are displayed in **Table 1**. PSMA RLT with [^161^Tb]Tb-PSMA-617 was administered under compassionate use provisions in accordance with section 13(2b) of the German Pharmaceutical Act. The decision to initiate the treatment was made on an individual basis in our multidisciplinary tumor board. Prior to treatment all patients gave written consent, following comprehensive information about potential risks and negative side effects of the interventions. Additionally, patients agreed to publication of any resulting data in anonymized form, in accordance with the Declaration of Helsinki. The study was approved by the local institutional review board (Ärztekammer des Saarlandes/Saarbrücken, ethics committee permission number 140/17).

### Radiolabeling and quality control

Radiolabeling of PSMA-617 with ^161^Tb, along with the quality control of [^161^Tb]Tb-PSMA-617, was carried out according to the published methodology for [^177^Lu]Lu-PSMA-617 27. For a standard labeling procedure of [^161^Tb]Tb-PSMA-617, ^161^Tb ([^161^Tb]TbCl₃ in 0.05 M HCl, TERTHERA B.V., Breda, Netherlands) was combined with a sodium acetate buffer (1.0 M, pH 4.5) containing PSMA-617 (Advanced Biochemical Compounds, ABX GmbH, Radeberg, Germany) to achieve a specific activity of approximately 42 MBq/nmol. The reaction mixture, adjusted to pH 4.5, was then incubated at 95 °C for 25 minutes. After cooling to ambient temperature, the reaction mixture was passed through a preconditioned C_18_ Sep Pak cartridge. The product was eluted with an ethanol/saline mixture (v/v 50:50) through a 0.22 µm sterile filter into a sterile product vial followed by formulation with saline. Quality control was performed using reversed-phase high-performance liquid chromatography (Shimadzu LC-20AT high-pressure liquid chromatography (HPLC) system). Radiochemical yields and purities were both ≥ 99% and the products were sterile and endotoxin free.

### Treatment details of [^161^Tb]Tb-PSMA-617 RLT

**Figure 1:**
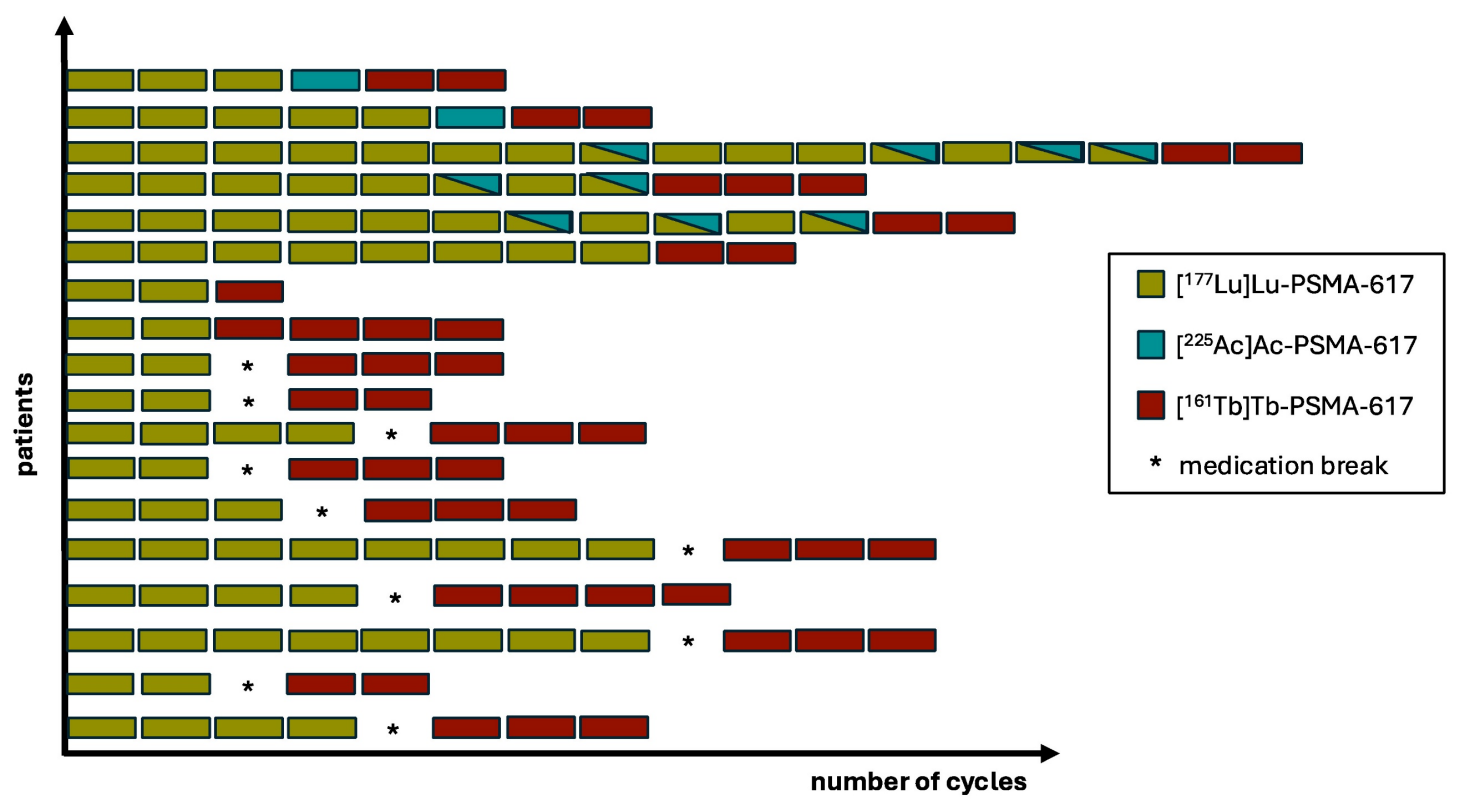
Regimen of PSMA RLT for each individual patient.

[^161^Tb]Tb-PSMA-617 RLT was performed with a median of 3 (range: 1 - 4) cycles; in total 47 cycles were administered to 18 patients. [^161^Tb]Tb-PSMA-617 RLT was initiated between 31^st^ August 2022 and 12^th^ July 2023. The mean administered activity of ^161^Tb per cycle was 6.2 ± 0.8 GBq (range: 3.1 - 9.1 GBq) and the mean cumulative activity was 16.1 ± 4.9 GBq (range: 5.9 - 23.2 GBq). The ^177^Lu activities recommended for non-compromised patients, as outlined in current guidelines 28, served as the initial reference point. Building upon this foundation, individualized dosing strategies were subsequently employed with the aim of optimizing therapeutic efficacy while minimizing associated risks. Administered activities were tailored on a per-patient basis, taking into account a range of individual clinical factors, including tumor burden, therapeutic urgency, extent of bone marrow involvement, disease progression, overall patient condition, and relevant hematologic parameters, as previously described by Khreish et al. 18. As prescribed by the German Radiation Protection Act, all patients were treated during an inpatient stay at our institution. All patients received cooling of the salivary glands during administration of the radioligand. Additionally, 30 min before injection, intravenous hydration was started (1000 mL 0.9% NaCl solution), which lasted until 120 min post-injection. The individual treatment regimen addressing the administered PSMA RLT cycles is presented in **Figure 1**.

### Response assessment

Biochemical and molecular imaging response was assessed. The biochemical response was evaluated via the measurement of serum PSA prior to [^161^Tb]Tb-PSMA-617 RLT (i.e. value at time of the first administration of [^161^Tb]Tb-PSMA-617) and follow-up values during and after [^161^Tb]Tb-PSMA-617 RLT. PSA levels were systematically assessed at each treatment cycle and follow-up in our department, as well as during outpatient visits by the respective treating oncologists or urologists, where applicable. For the subsequent analysis, the best PSA response observed after initiation of [^161^Tb]Tb-PSMA-617 RLT was used.

Progressive disease (PD) was defined as a PSA value increasing ≥ 25% from baseline to follow-up 29. Partial remission (PR) of disease was defined as the baseline PSA value decreasing ≥ 50% (PSA50 threshold), and stable disease (SD) was defined as a PSA decrease of < 50% or an increase < 25%. All patients who achieved PR were classified as responders, while those presenting SD or PD were categorized as non-responders to therapy. Molecular imaging response was evaluated by [^68^Ga]Ga-PSMA-11 PET/CT. All patients received [^68^Ga]Ga-PSMA-11 PET/CT at baseline and follow up. Baseline PSMA PET/CT was performed a mean of 2 ± 2 weeks prior to the first [^161^Tb]Tb-PSMA-617 RLT cycle, and follow-up imaging was conducted a mean of 5 ± 2 weeks after.

In accordance with the guidelines for prostate cancer imaging 30, the interval between tracer injection and imaging was 60 minutes. All PET/CT scans were conducted using a Biograph 40 mCT PET/CT scanner (Siemens Medical Solutions, Knoxville, TN, USA). The acquisition time was 3 minutes per bed position, with a slice thickness of 3.00 mm, and an extended field of view of 21.4 cm (TrueV) was applied. For attenuation correction and anatomical localization, a low-dose CT scan was acquired. PET image reconstruction was performed using a three-dimensional OSEM algorithm with 3 iterations, 24 subsets, Gaussian filtering, and a slice thickness of 5.00 mm. Based on these scans, total lesion PSMA (TLP) values 31 representing total tumor burden were measured by semi-automated tumor segmentation algorithm using the Syngo.Via software (Enterprise VB 40B, Siemens, Erlangen, Germany). TLP is quantified as the cumulative product of lesion volume and mean standardized uptake value (SUV_mean_) across all identified lesions (∑ Volume × SUV_mean_). For delineation, standardized uptake value (SUV) ≥ 3 was used as a threshold, following the description by Ferdinandus et al. 32. Lesions with a volume < 0.2 mL were automatically excluded. Physiological uptake such as in the liver, spleen, bladder, or salivary glands was manually excluded. Based on the change of TLP values, patients were categorized as follows: PD was defined as a TLP increase ≥ 30%, PR was defined as a decrease ≥ 30% and SD was defined as an increase of TLP < 30% or a decrease < 30%.

### Adverse events

In accordance with the Common Terminology Criteria for Adverse Events (CTCAE v5.0; https://ctep.cancer.gov/protocoldevelopment/electronic_applications/docs/CTCAE_v5_Quick_Reference_8.5x11.pdf; last accessed 26^th^ May 2025), adverse events were graded within the registry at both baseline and each follow-up visit. The evaluation particularly focused on hematologic toxicities—namely anemia, leukopenia, and thrombocytopenia—based on blood count analyses. Renal toxicity was evaluated via calculation of creatinine-based estimated glomerular filtration rate (eGFR), while xerostomia, fatigue and nausea were rated applying a CTCAE-based questionnaire completed by the patient. Details regarding the assessment of fatigue and xerostomia are presented in the supplementary material (**Table S2**).

### Statistical analysis

Descriptive statistical analyses were conducted using PRISM 10 (GraphPad Software, San Diego, USA). The analysis of survival data was conducted using the Kaplan-Meier method. Progression-free survival (PFS) was defined as the time between the commencement of [^161^Tb]Tb-PSMA-617 RLT and the occurrence of one of the following events: detection of biochemical progression, death or the last study-visit (censored). Overall survival (OS) was defined as the time elapsed between the commencement of [^161^Tb]Tb-PSMA-617 RLT and the occurrence of either death or the last study visit (censored). Follow up time was calculated using inverse Kaplan-Meier method.

## Results

Response assessment was performed biochemically and through molecular imaging using either the change of serum PSA or the course of the total tumor biomarker TLP. The mean baseline PSA value prior to the first [^161^Tb]Tb-PSMA-617 cycle was 138.0 ± 144.6 ng/mL (range: 0.4 - 474 ng/mL), while the mean (best) follow-up value was 107.7 ± 121.0 ng/mL (range: 0.2 - 376 ng/mL), equaling a mean PSA decrease (ΔPSA) of -22.0% (95% CI: -51.2 - +2.1 %) after initiation of [^161^Tb]Tb-PSMA-617 RLT. The mean baseline TLP value before initiation of [^161^Tb]Tb-PSMA-617 RLT was 3435 ± 4953 SUV x mL (range: 58.3 - 17550), compared to a mean follow-up value of 3129 ± 4763 SUV x mL (range: 7.9 - 14545), with a mean decrease of -25.9% (95% CI: -50.4 - -1.5 %).

**Figure 2:**
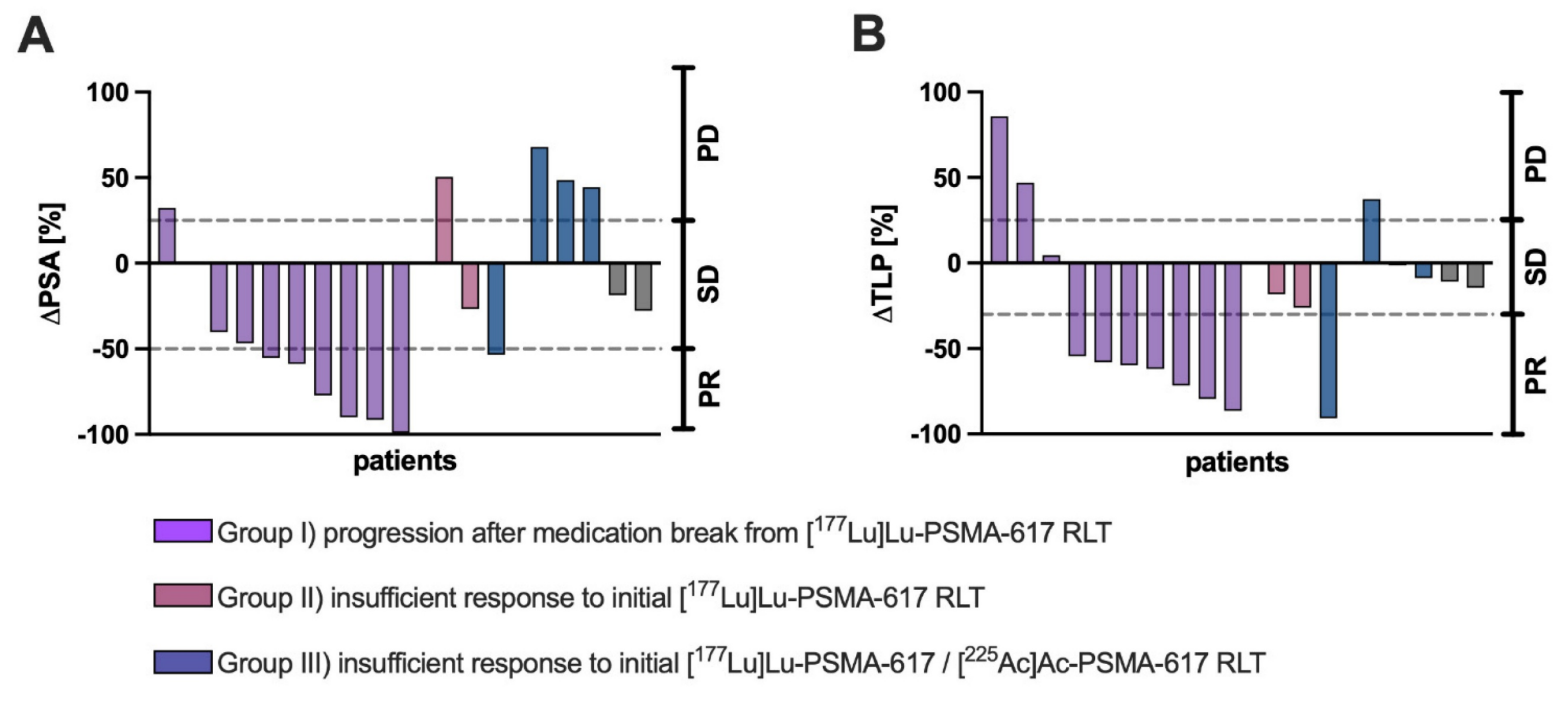
Individual **(A)** ΔPSA (%) and **(B)** ΔTLP (%) values of each patient receving [^161^Tb]Tb-PSMA-617 RLT with categorization according to different clinical settings (purple: progression after medication break from [^177^Lu]Lu-PSMA-617 RLT, red: insufficient response to initial [^177^Lu]Lu-PSMA-617 RLT, blue: insufficient response to [^225^Ac]Ac-PSMA-617 augmented [^177^Lu]Lu-PSMA-617 RLT).

Individual ΔPSA (%) and ΔTLP (%) for each patient with categorization according to different clinical settings are presented in **Figure 2**. A patient individual overview presenting ΔPSA, ΔTLP and the respective response category is presented in the supplementary material (**Table S3**).

**Figure 3:**
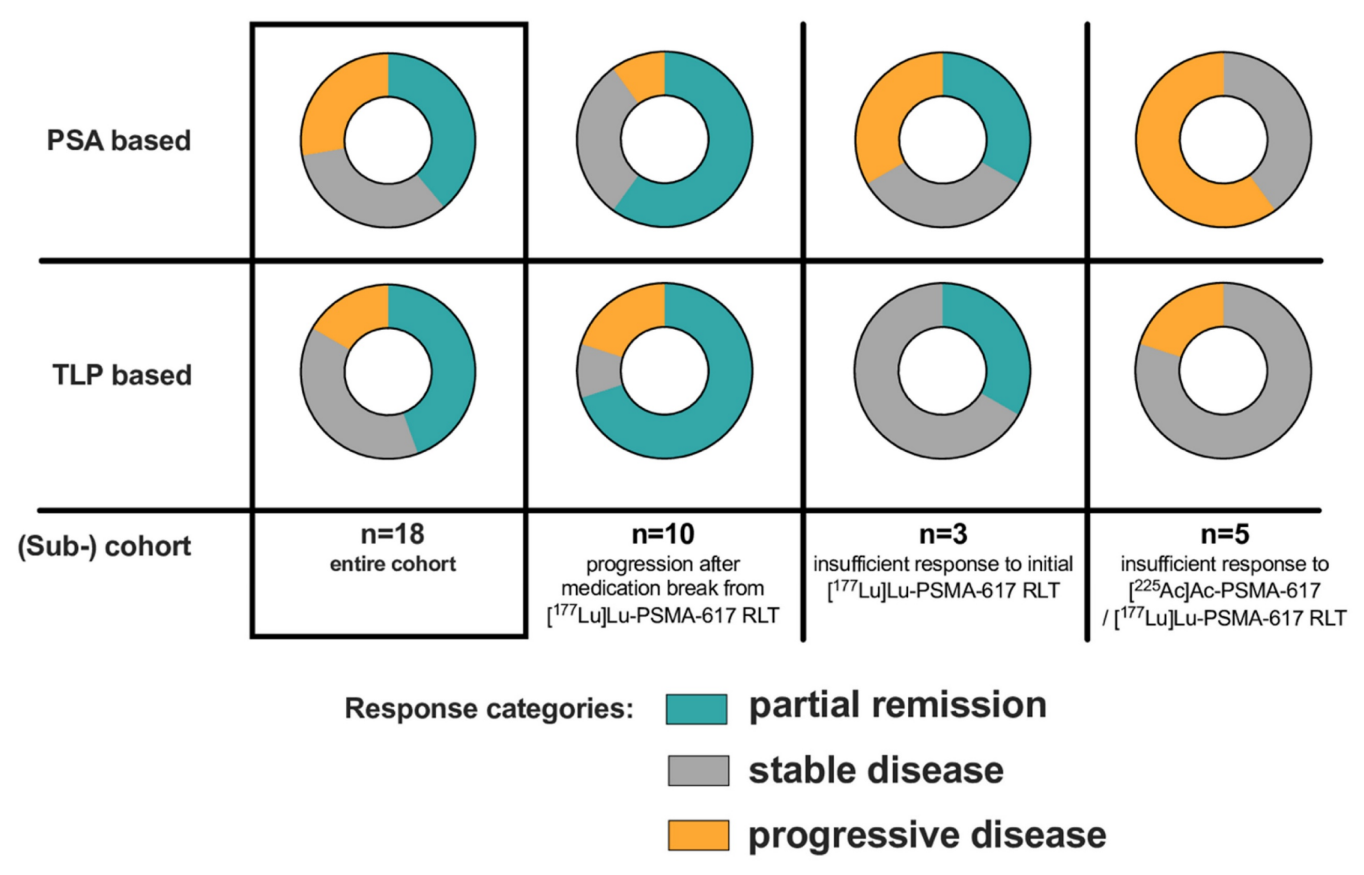
Summary of PSA- and TLP-based response for the entire cohort and categorized according to the different clinical settings.

7/18 patients (38.9%) of the entire cohort were biochemical (PSA50) responders (PR), whereas 8/18 patients (44.4%) exhibited PR based on molecular imaging (TLP, **Figure 3**).

**Figure 4:**
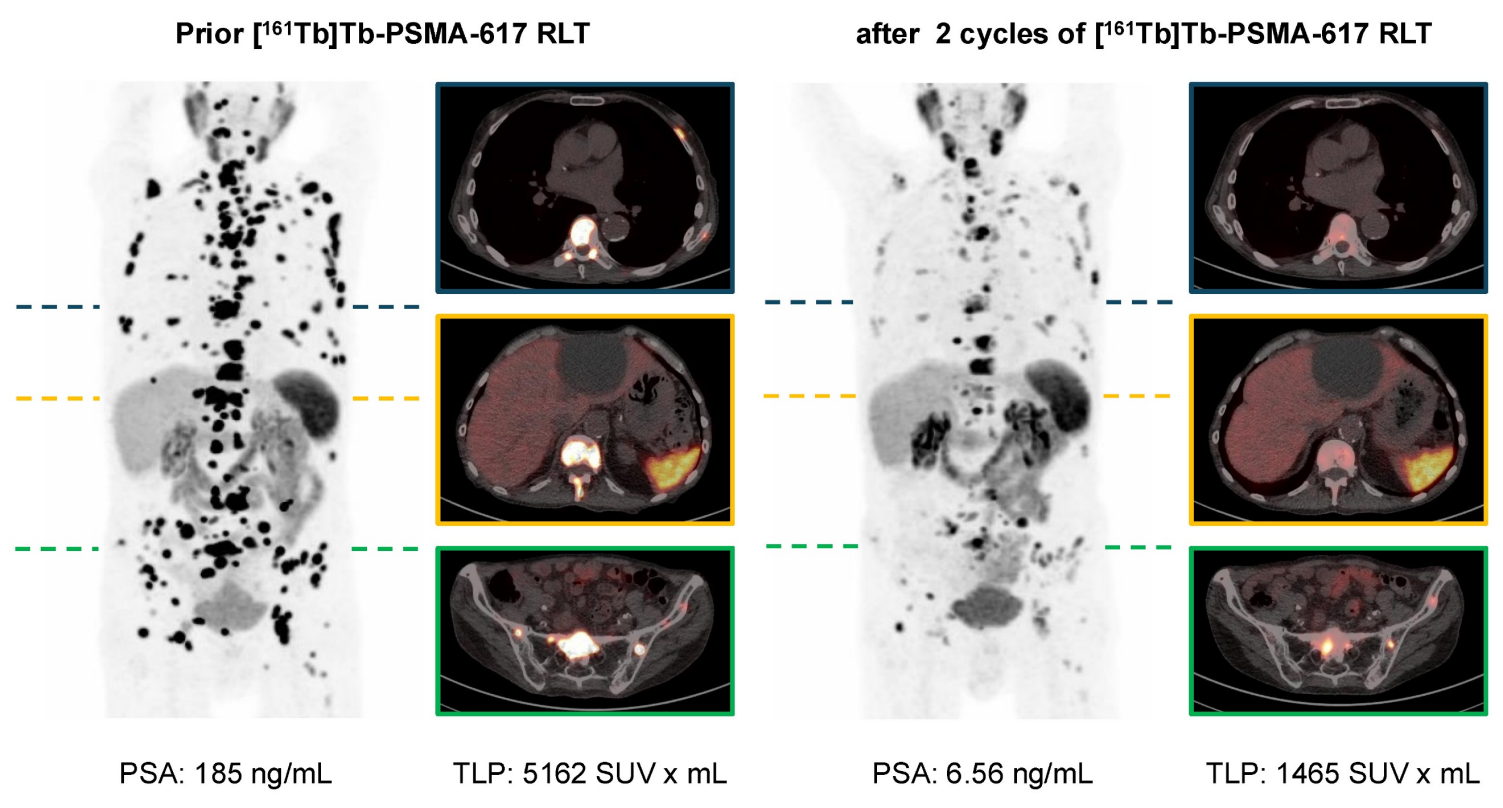
[^68^Ga]Ga-PSMA-11 PET/CT with maximum intensity projections (MIP) and transversal PET/CT slices of a patient with mCRPC undergoing [^161^Tb]Tb-PSMA-617 RLT. The patient experienced post-RLT progression after [^177^Lu]Lu-PSMA-617 RLT (initial 4 cycles); subsequently [^161^Tb]Tb-PSMA-617 RLT was initiated resulting in biochemical and molecular imaging partial remission (PR).

In the clinical setting of post-RLT progression after initial response to [^177^Lu]Lu-PSMA-617 RLT (n = 10), 6/10 (60%) and 7/10 (70%) showed PR by both biochemical and molecular imaging assessment after receiving [^161^Tb]Tb-PSMA-617. SD and PD were noted in 3/10 (30%) and 1/10 (10%) patients in biochemical assessment and in 1/10 (10%) and 2/10 (20%) patients in molecular imaging assessment, respectively. An exemplary patient experiencing biochemical and molecular imaging PR is presented in **Figure 4**.

For the clinical setting of insufficient response to initial [^177^Lu]Lu-PSMA-617 RLT (n=3), 1/3 (33.3%) patients had PR on both assessment methods. By ΔPSA, 1 patient (33.3%) experienced SD and 1 patient (33.3%) PD; by molecular imaging 2 patients (66.6%) exhibited SD.

In the clinical setting of insufficient response to [^225^Ac]Ac-PSMA-617 augmented [^177^Lu]Lu-PSMA-617 RLT no patient experienced PR, neither by ΔPSA nor by ΔTLP. 3/5 patients (60%) were biochemically categorized as PD, while 2/5 patients (40%) were evaluated as SD. By molecular imaging marker TLP, 4/5 patients (80%) experienced SD and 1/5 patients (20%) showed PD.

**Figure 5:**
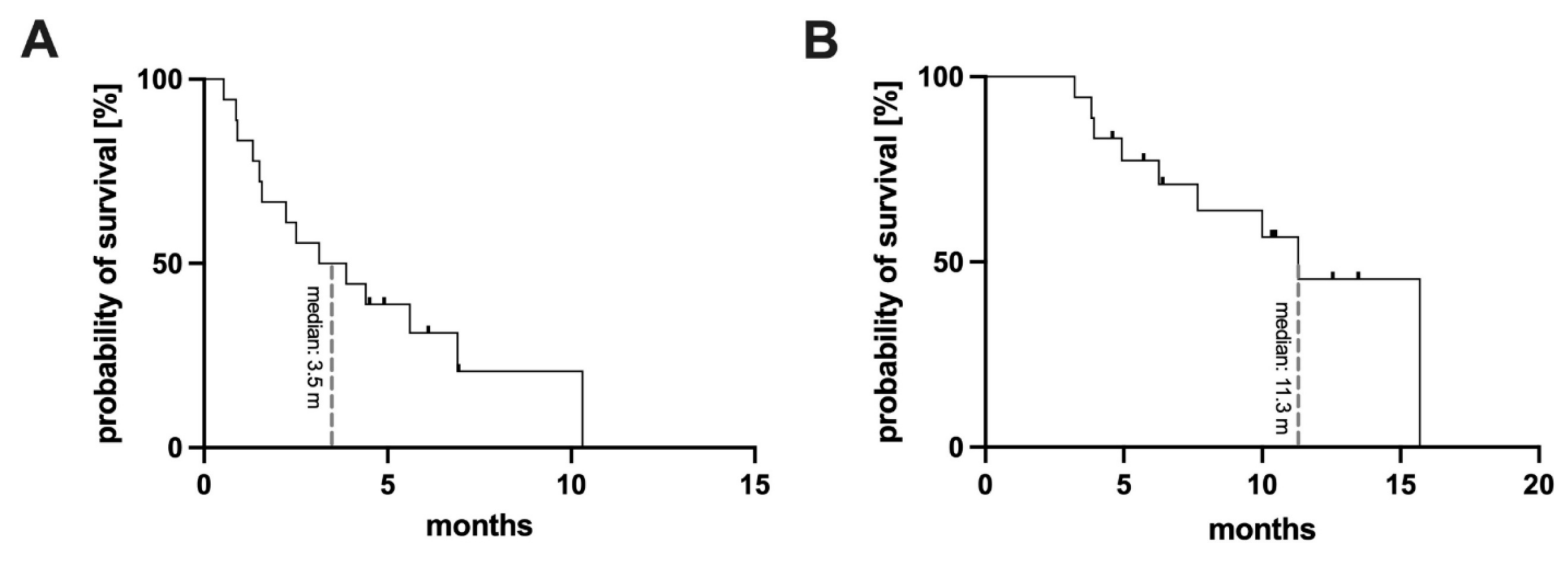
Kaplan-Meier curves for the entire cohort, presenting progression-free survival **(A)** and overall survival **(B)**.

For the whole cohort the median follow up time was 10.5 months. Starting from commencement of [^161^Tb]Tb-PSMA-617 RLT (the first cycle of [^161^Tb]Tb-PSMA-617), a median PFS of 3.5 months (95% CI: 0.3 - 6.0 months) and a median OS of 11.3 months (95% CI: 8.5 -14.1 months) was found. The respective Kaplan-Meier curves are presented in **Figure 5**. Analysis of the different clinical settings revealed: (i) patients with progression following initial PSMA RLT showed PFS of 6.9 months (95% CI: 4.3 - 9.5 months), and median OS was not reached at date of analysis (median follow up time 10.2 months), (ii) patients with insufficient response to initial PSMA RLT presented a PFS of 1.5 months (95% CI: 1.2 - 1.8 months), and median OS of 11.3 months (95% CI: 0.0 - 23.1 months), and (iii) patients with insufficient response to [^225^Ac]Ac-PSMA-617 augmented [^177^Lu]Lu-PSMA-617 RLT exhibited a PFS and OS of 0.9 months (95% CI: 0.8 - 1.0 months) and 7.7 months (95% CI: 4.7 - 10.7 months), respectively, under [^161^Tb]Tb-PSMA-617 RLT*.*

**Figure 6:**
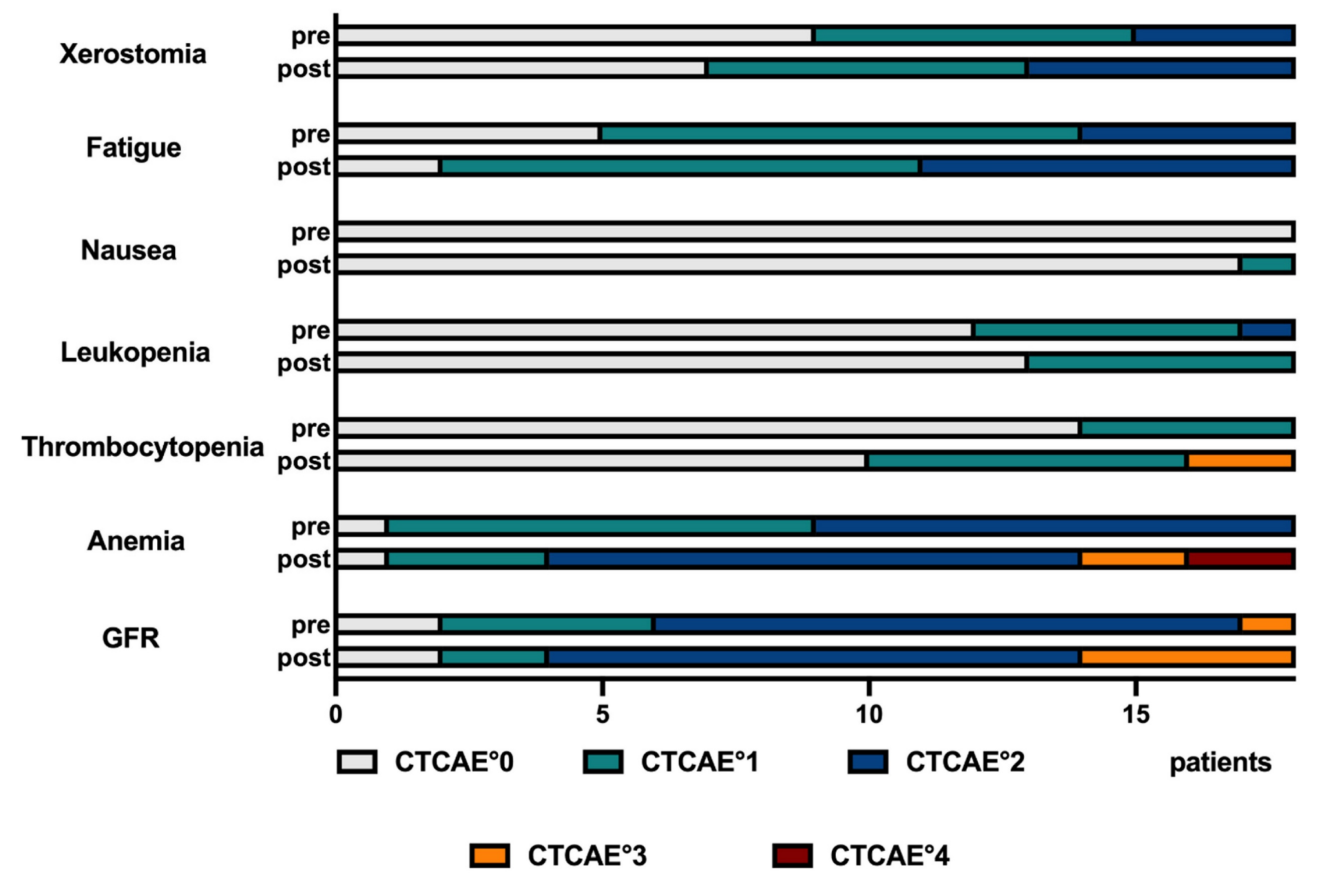
Record of adverse events for the entire cohort, following the CTCAE grading system.

The majority of all recorded adverse events was rated mild or moderate (CTCAE °1 or °2); higher grades (CTCAE °3 or °4) were rarely observed. The details of CTCAE gradings are presented in **Figure 6,** and for each individual patient in the supplemental material (**Table S4**). In detail, 8 CTCAE deteriorations from CTCAE °2 to °3 or from °3 to °4 were observed: 2 of post-therapeutic CTCAE °3 thrombocytopenia, 2 post-therapeutic anemia °3 and 4 post-therapeutic renal impairment °3 occurred. Additionally, 2 patients showed a deterioration to °4 anemia during therapy, starting from a pre-therapeutic CTCAE °2. However, no termination of PSMA RLT due to occurring side effects was recorded.

## Discussion

PSMA targeted radioligand therapy with ^177^Lu has revolutionized the treatment of mCRPC. However, after disease progression, there is still an unmet need for further treatment options. ^161^Tb is an emerging and increasingly discussed radionuclide, showing potential as a candidate for targeted radionuclide therapy. By emitting a considerable amount of additional low-energy Auger and conversion electrons, ^161^Tb offers physical advantages over the commonly used ^177^Lu, resulting in higher local absorbed doses. In this study, we present initial experience with [^161^Tb]Tb-PSMA-617 RLT from patients participating in a prospective registry (NCT04833517) across different clinical settings following initial standard PSMA RLT.

Our results suggest that [^161^Tb]Tb-PSMA-617 RLT is a feasible treatment option following conventional PSMA RLT. Overall, we observed a promising response rate around 40% (PSA50 - rated as biochemical PR - in 38.9% and molecular imaging PR in 44.4% of patients), with a median PFS of 3.5 months and OS of 11.3 months, respectively, in a heterogeneous cohort of patients previously experiencing insufficient response or renewed progression after initial RLT (which was even augmented with [^225^Ac]Ac-PSMA-617 in 27.8 % of patients). To the best of our knowledge, with a sample size of n = 18 patients and 47 cycles, this study is, to date, the largest experience on [^161^Tb]Tb-PSMA-617 RLT and notably the only study in non-RLT-naïve patients with mCRPC. Most published work on ^161^Tb is preclinical but has already demonstrated, via cellular assays and murine models, that ^161^Tb is promising and superior to ^177^Lu in terms of antitumor effects 33-36. Dosimetric simulations and calculations have also suggested such superiority, especially for micro-metastases 37,38. However, published clinical applications of ^161^Tb are scarce and primarily descriptive. There are individual case reports and small subcohorts for the use of ^161^Tb in PSMA RLT 39-43 and other forms of radionuclide therapy such as peptide radioreceptor therapy (PRRT) 44,45. In a pilot study involving n=6 patients with mCRPC, we were able to demonstrate the dosimetric superiority of ^161^Tb in a head-to-head in-vivo comparison to ^177^Lu, observing a 2.4-fold higher dose in tumor lesions, with only a slight increase in the dose to normal organs 46. The current study focuses on the outcome and safety of [^161^Tb]Tb-PSMA-617 RLT in patients following initial PSMA RLT.

In detail, the settings in which ^161^Tb was applied in this study included: (i) patients with renewed progression after conventional PSMA RLT (ii) patients with insufficient response to conventional PSMA RLT, and (iii) patients with progression under [^225^Ac]Ac-PSMA-617 augmented [^177^Lu]Lu-PSMA-617 RLT. The subgroups showed varying outcomes. The best response was observed in the group of patients who experienced post-RLT progression after [^177^Lu]Lu-PSMA-617 RLT. This may be explained by preserved tumor sensitivity to beta radiation and suggests ^161^Tb as a potential alternative to conventional ^177^Lu based PSMA RLT, exploiting the advantages of the high proportion of low-energy conversion and Auger electrons. Further studies are needed to investigate [^161^Tb]Tb-PSMA-617 in the context of earlier stages of CRPC or even HSPC as well as to analyze [^161^Tb]Tb-PSMA-617 in comparison to [^177^Lu]Lu-PSMA-617 for rechallenge RLT where benchmark data already exists 47-49.

While ^161^Tb may potentially allow escalating conventional [^177^Lu]Lu-PSMA-617 RLT as also described in previous case reports 39,40, no patient who had previously progressed under [^225^Ac]Ac-PSMA-617 augmented [^177^Lu]Lu-PSMA-617 RLT responded to [^161^Tb]Tb-PSMA-617 RLT in our current study. This suggests that ^161^Tb alone may not be used for potentially escalating RLT after ^225^Ac, not unexpectedly in view of the physical properties.

Regarding the adverse events of [^161^Tb]Tb-PSMA-617 treatment, the study found that the side effect profile was mostly comparable to that observed with [^177^Lu]Lu-PSMA-617 RLT 15,18. Adverse events graded as °3 or °4 were rare, and no treatment discontinuation due to severe side effects was required. Overall, the results indicate no disadvantage of ^161^Tb over ^177^Lu in terms of potential side effects.

In summary, the results support the potential of ^161^Tb as a promising radionuclide for PSMA-targeted radioligand therapy. However, it is important to note that the large-scale availability of ^161^Tb, comparable to that of currently standard used ^177^Lu, remains open. The future development of global production capacity, worldwide availability constraints, and logistical challenges will be critical factors for its clinical translation.

It is essential to acknowledge the limitations of this study, including its retrospective and single-center design. Also, the small sample size and the varying sizes of the sub-cohorts might be a potential source of selection bias (towards responders to initial RLT) and limits the statistical power and comparability. Moreover, no standardized activity protocol was employed, and the variability in individual activities may have influenced the outcome. Another limitation might be lack of performed [^18^F]FDG PET/CT before patient selection as well as an infrequent performed post-therapeutic SPECT/CT imaging. Biochemical profile analysis only focused on PSA values, small blood sample and renal function, subsequent studies should also analyze further values e.g. ALP, LDH and liver function parameters.

Future studies, ideally in larger prospective cohorts, are needed to confirm and expand upon these findings. A first international prospective study - the Phase I/II VIOLET study (NCT05521412) - for [^161^Tb]Tb-PSMA-I&T has been initiated and is underway in Australia 50. The data presented here could serve as a rationale and starting point for further prospective investigations.

## Conclusion

These pilot results confirm ^161^Tb as a promising radionuclide for PSMA RLT and suggest [^161^Tb]Tb-PSMA-617 as a potential effective and safe treatment option even in the advanced mCRPC setting after multi-line systemic therapies including standard PSMA RLT. Larger studies are warranted to confirm and extend this initial experience and clinical trials in earlier stages of CRPC appear promising based on our initial impression of this radionuclide-based novelty in PSMA RLT.

## Supplementary Material

Supplementary tables.

## Figures and Tables

**Table 1:** Patient characteristics

Patient characteristics	Value
**Age**	
Median (range)	76 (65 - 87)
Age ≥ 65 years, n (%)	18 (100)
**PSA,** in [ng/mL]	
Median (range)	90 (0.4 - 474)
**ALP,** in [U/L]	
Median (range)	100 (24 - 449)
**Hemoglobin,** in [g/dL]	
Median (range)	10 (8 - 14)
< 13 g/dL, n (%)	16 (88.9)
**ECOG performance status,** n (%)	
0	2 (11.1)
1	14 (77.8)
≥2	2 (11.1)
**Sites of metastases,** n (%)	
Bone	15 (83.3)
Lymph node	12 (66.7)
Liver	2 (11.1)
Other	5 (27.8)
**Prior therapies,** n (%)	
Prostatectomy	8 (44.4)
Radiation	14 (77.8)
ADT	18 (100)
NAAD	17 (94.4)
Abiraterone	14 (77.8)
Enzalutamide	17 (94.4)
Abiraterone and Enzalutamide	14 (77.8)
Chemotherapy	15 (83.3)
Docetaxel	15 (83.3)
Cabazitaxel	5 (27.8)
Docetaxel and Cabazitaxel	5 (27.8)
^223^Ra-dichloride	5 (27.8)
PSMA RLT	18 (100)
^177^Lu-PSMA-617 RLT	18 (100)
^225^Ac augmented ^177^Lu-PSMA-617 RLT	5 (27.8)
Prior PSMA RLT cycles, median (range)	
^177^Lu-PSMA-617	4 (2 - 15)
Median activity per cycle in [GBq] (range)	7.3 (3.3 - 9.3)
Median cumulative activity in [GBq] (range)	26.7 (14 - 101.5)
^225^Ac-PSMA-617	2 (1 - 4)
Median activity per cycle in [MBq] (range)	4.2 (2.2 - 10)
Median cumulative activity in [MBq] (range)	6.3 (2.8 - 19.2)

ADT = androgen deprivation therapy; ALP = alkaline phosphatase; ECOG = Eastern Cooperative Oncology Group; NAAD = novel androgen axis drugs; PSA = prostate-specific antigen; PSMA RLT = prostate-specific membrane antigen targeted radioligand therapy.
