# Supplementary material for: Pilot experience of [161Tb]Tb-PSMA-617 RLT in mCRPC patients after conventional PSMA RLT within a prospective registry
Source: Theranostics. 2025 Aug 16;15(17):9019–28. doi: 10.7150/thno.115831 (PMC12439265; doi:10.7150/thno.115831)
Supplement: Supplementary file 1 — Supplementary tables. [file thnov15p9019s1.pdf]

Pilot experience of [^{161}Tb]Tb-PSMA-617 RLT in mCRPC patients after conventional PSMA RLT within a prospective registry

Florian Rosar[1], Caroline Burgard[1], Christine Petrescu[1], Arne Blickle[1], Mark Bartholomä[1], Stephan Maus[1], Moritz B. Bastian[1], Tilman Speicher[1], Andrea Schaefer-Schuler[1] and Samer Ezziddin[1]

1 Department of Nuclear Medicine, Saarland University – Medical Center, Homburg, Germany

Corresponding authors e-mail: samer.ezziddin@uks.eu

Table S1: Physical properties of the nuclides ^{161}Tb and ^{177}Lu based on data from the *International Commission on Radiological Protection (ICRP)* [1].

Parameter	^{161}Tb	^{177}Lu
**Physical half-life** [days]	6.906	6.647
**Decay mode**	β^- (100%)	β^- (100%)
**Stable daughter nuclide**	^{161}Dy	^{177}Hf
**Mean β^- energy** [keV]	154.3	133.3
**Total electron energy per decay** [keV]	202.5	147.9
**Conversion electron energy per decay** [keV]	39.28	13.52
**Conversion electron spectrum** [keV] (weighted average mean)	3.3 – 98.3 (28)	6.2 – 206.3 (87)
**Auger electron energy per decay** [keV]	8.94	1.13
**Auger electron spectrum** [keV] (weighted mean)	0.018 – 50.9 (0.8)	0.01 – 61.7 (1)
**Imaging relevant γ emissions [keV]** ([%] abundance)	75 (10.2%)	208 (10.4%)/ 113 (6.4%)

Table S2: Grading system of fatigue and xerostomia after the of the U.S. Department of Health and Human Services (National Institutes of Health – National Cancer Institute).

CTCAE Term	Grade 1	Grade 2	Grade 3	Grade 4	Grade 5
**Fatigue**	Fatigue relieved by rest.	Fatigue not relieved by rest; limiting instrumental activities of daily living.	Fatigue not relieved by rest; limiting self care activities of daily living.	X	X
**Definition:** A disorder characterized by a state of generalized weakness with a pronounced inability to summon sufficient energy to accomplish daily activities.					
**Xerostomia**	Symptomatic (e.g., dry or thick saliva) without significant dietary alteration.	Moderate symptoms; oral intake alterations (e.g., copious water, other lubricants, diet limited to purees and/or soft, moist foods).	Inability to adequately aliment orally; tube feeding or TPN indicated.	X	X
**Definition:** A disorder characterized by reduced salivary flow in the oral cavity.					

Table S3: Patient individual ΔPSA value, ΔTLP value and response category after treatment with [^{161}Tb]Tb-PSMA-617.

Patient	Subgroup (I/II/III)	ΔPSA [%]	ΔTLP [%]	Response Category (ΔPSA based)	Response Category (ΔTLP based)
**P1**	I	-40.2	-54.5	SD	PR
**P2**	I	-89.9	-86.4	PR	PR
**P3**	I	-55.3	-59.8	PR	PR
**P4**	I	-99.0	-71.6	PR	PR
**P5**	I	-46.7	-57.9	SD	PR
**P6**	I	-58.8	-62.0	PR	PR
**P7**	I	-92.2	-79.4	PR	PR
**P8**	I	-77.3	47.0	PR	PD
**P9**	I	32.3	85.8	PD	PD
**P10**	I	-0.9	4.66	SD	SD
**P11**	II	-26.8	-26.2	SD	SD
**P12**	II	50.5	-18.2	PD	SD
**P13**	II	-53.4	-90.8	PR	PR
**P14**	III	-18.6	-14.4	SD	SD
**P15**	III	68.0	-8.7	PD	SD
**P16**	III	48.6	37.4	PD	PD
**P17**	III	44.4	-1.4	PD	SD
**P18**	III	-27.8	-10.7	SD	SD

I: progression after medication break from [^{177}Lu]Lu-PSMA-617 RLT; II: insufficient response to initial [^{177}Lu]Lu-PSMA-617 RLT and III: insufficient response to initial [^{177}Lu]Lu-PSMA-617/[^{225}Ac]Ac-PSMA-617 RLT

Table S4: Course of CTCAE gradings of the individual patients during PSMA RLT.

	Before start of initial PSMA RLT						
Patient	Xerostomie (CTCAE)	Fatigue (CTCAE)	Nausea (CTCAE)	Leucopenia (CTCAE)	Thrombo-cytopenia (CTCAE)	Anemia (CTCAE)	GFR (CTCAE)
**P1**	0	1	0	0	0	0	0
**P2**	0	1	0	0	1	1	2
**P3**	0	0	0	1	0	1	0
**P4**	0	1	0	0	0	2	2
**P5**	1	0	0	0	0	1	1
**P6**	0	0	0	0	0	0	2
**P7**	0	1	0	0	0	1	0
**P8**	0	1	0	0	0	2	2
**P9**	0	1	0	0	0	1	1
**P10**	0	1	0	0	0	1	2
**P11**	0	1	0	0	0	3	1
**P12**	0	0	0	0	0	1	1
**P13**	0	0	0	0	0	2	1
**P14**	0	0	0	0	0	1	1
**P15**	0	0	0	0	0	1	2
**P16**	0	1	0	0	0	1	1
**P17**	2	2	0	0	1	3	0
**P18**	0	1	0	1	0	1	2
	**Before start of [^{161}Tb]Tb-PSMA-617 RLT**						
Patient	Xerostomie (CTCAE)	Fatigue (CTCAE)	Nausea (CTCAE)	Leucopenia (CTCAE)	Thrombo-cytopenia (CTCAE)	Anemia (CTCAE)	GFR (CTCAE)
**P1**	0	1	0	0	0	0	0
**P2**	1	1	0	0	0	1	2
**P3**	2	1	0	1	0	1	1
**P4**	0	0	0	0	0	2	2
**P5**	0	1	0	1	0	1	2
**P6**	1	0	0	1	1	1	2
**P7**	0	0	0	0	0	1	1
**P8**	0	0	0	0	1	2	3
**P9**	1	1	0	0	0	1	2
**P10**	0	2	0	0	0	1	2
**P11**	0	1	0	1	1	2	1
**P12**	0	0	0	0	0	2	1
**P13**	1	2	0	0	0	2	2
**P14**	2	1	0	0	0	1	2
**P15**	2	2	0	0	0	2	2
**P16**	1	1	0	0	0	2	2
**P17**	1	2	0	2	1	2	0
**P18**	0	1	0	1	0	2	2
	**After [^{161}Tb]Tb-PSMA-617 RLT**						
Patient	Xerostomie (CTCAE)	Fatigue (CTCAE)	Nausea (CTCAE)	Leucopenia (CTCAE)	Thrombo-cytopenia (CTCAE)	Anemia (CTCAE)	GFR (CTCAE)
**P1**	0	1	0	0	0	0	1
**P2**	0	1	0	0	0	1	2
**P3**	2	1	0	1	1	2	2
**P4**	1	2	0	0	0	2	3
**P5**	0	2	0	1	1	2	2
**P6**	1	0	0	0	1	1	2

**P7**	0	1	0	0	0	1	0
**P8**	0	0	0	0	1	2	2
**P9**	1	1	0	0	0	3	3
**P10**	0	2	0	0	0	2	2
**P11**	1	2	0	0	3	3	1
**P12**	0	1	0	0	0	2	2
**P13**	1	2	0	0	0	4	2
**P14**	2	1	0	0	1	2	3
**P15**	2	2	0	0	1	2	3
**P16**	2	1	0	1	0	4	2
**P17**	2	2	1	1	3	2	0
**P18**	1	1	0	1	0	2	2

References

1. Eckerman K, Endo A. ICRP Publication 107. Nuclear decay data for dosimetric calculations. Ann ICRP. 2008; 38: 7–96.
